# Comparison of Amino Acids Physico-Chemical Properties and Usage of Late Embryogenesis Abundant Proteins, Hydrophilins and WHy Domain

**DOI:** 10.1371/journal.pone.0109570

**Published:** 2014-10-08

**Authors:** Emmanuel Jaspard, Gilles Hunault

**Affiliations:** 1 Université d'Angers, UMR 1345 IRHS, SFR 4207 QUASAV, Angers, France; 2 INRA, UMR 1345 IRHS, Beaucouzé, France; 3 Agrocampus-Ouest, UMR 1345 IRHS, Angers, France; 4 Université d'Angers, Laboratoire d'Hémodynamique, Interaction Fibrose et Invasivité tumorale hépatique, UPRES 3859, IFR 132, F-49045 Angers, France; University of Cincinnati, United States of America

## Abstract

Late Embryogenesis Abundant proteins (LEAPs) comprise several diverse protein families and are mostly involved in stress tolerance. Most of LEAPs are intrinsically disordered and thus poorly functionally characterized. LEAPs have been classified and a large number of their physico-chemical properties have been statistically analyzed. LEAPs were previously proposed to be a subset of a very wide family of proteins called hydrophilins, while a domain called WHy (Water stress and Hypersensitive response) was found in LEAP class 8 (according to our previous classification). Since little is known about hydrophilins and WHy domain, the cross-analysis of their amino acids physico-chemical properties and amino acids usage together with those of LEAPs helps to describe some of their structural features and to make hypothesis about their function. Physico-chemical properties of hydrophilins and WHy domain strongly suggest their role in dehydration tolerance, probably by interacting with water and small polar molecules. The computational analysis reveals that LEAP class 8 and hydrophilins are distinct protein families and that not all LEAPs are a protein subset of hydrophilins family as proposed earlier. Hydrophilins seem related to LEAP class 2 (also called dehydrins) and to Heat Shock Proteins 12 (HSP12). Hydrophilins are likely unstructured proteins while WHy domain is structured. LEAP class 2, hydrophilins and WHy domain are thus proposed to share a common physiological role by interacting with water or other polar/charged small molecules, hence contributing to dehydration tolerance.

## Introduction

Some organisms can survive the almost total loss of their cellular water in a process that is called anhydrobiosis. The most common anhydrobiotes are found in higher plants, since in most species, orthodox seeds acquire desiccation tolerance during maturation. Once shed as dry and quiescent organisms, seeds can be stored for very long periods before resuming life during imbibition, and rapidly germinate. Considering the constraint imposed by desiccation to biological structures and components, it is not surprising that specific proteins are expressed in the context of anhydrobiosis. LEAPs were originally discovered in *Gossypium hirsutum* seeds [1]–[5]. They are especially prominent in plants with up to 71 genes annotated as LEAP in *Arabidopsis* [6]–[8]. LEAPs have been also identified in bacteria, fungi, algae and animals [9]–[12] and are associated with abiotic stress tolerance, particularly dehydration, cold stress and salt stress [3], [13]–[15] suggesting a general protective role in anhydrobiotic organisms.

Most of LEAPs are intrinsically disordered proteins (IDP) and thus little is known about their molecular mechanism of action, although *in vitro* assays with various LEAPs suggested roles in desiccation and/or freezing aggregation [16], [17] or membrane protection [18]–[20]. For example, *in vitro* experiments have shown that in the hydrated state, mitochondrial LEAP is unfolded and does not hamper mitochondrial functioning, while in the dry state, it folds and enters the inner membrane to provide protection [19]–[21]. LEAPs were also shown to sequester calcium [22], metal ions [23] and reactive oxygen species [24] and to contribute to the glassy state [25].

However, despite their role in membrane protection and some theoretical studies such as molecular dynamics simulations [10] the actual functional mechanism of LEAPs at the molecular level remains to be demonstrated for most of them.

Investigating the structure - function relationships of LEAPs is thus of primary interest, but remains challenging because experimental evidence is difficult to obtain. A database called LEAPdb (http://forge.info.univ-angers.fr/~gh/Leadb/index.php) dedicated to this purpose is available [8] and LEAPs have been classified in 12 non-overlapping classes. A large number of physico-chemical properties of the LEAP classes have been computed and statistically analyzed [26].

Since LEAPs were early recognized as highly hydrophilic proteins, this led Garay-Arroyo *et al*. [27] to propose they were members of a more widespread group of proteins, which they coined hydrophilin, characterized by a high glycine content and high average hydrophilicity. Interestingly, in yeast and *Escherichia coli*, hydrophilins expression appeared well correlated with osmotic stress [27], [28] and the yeast hydrophilin STF2p was found to be essential for dehydration tolerance [29]. In a further analysis, in which the Gly criteria for hydrophilins was lowered to 6%, Battaglia *et al*. [30] concluded that LEAPs were indeed hydrophilins since 92% of 378 LEAPs fulfilled a high Gly content and a low hydrophobicity.

Water stress and hypersensitive response (WHy) domain is a region of unknown function found in several plant proteins involved in either the response to water stress or the response to bacterial infection [31]. WHy domain is also found in several bacterial and archaeal proteins whose functions are not currently known. WHy domain was identified as a signature of LEAP class 8 [8].

**Figure 1:**
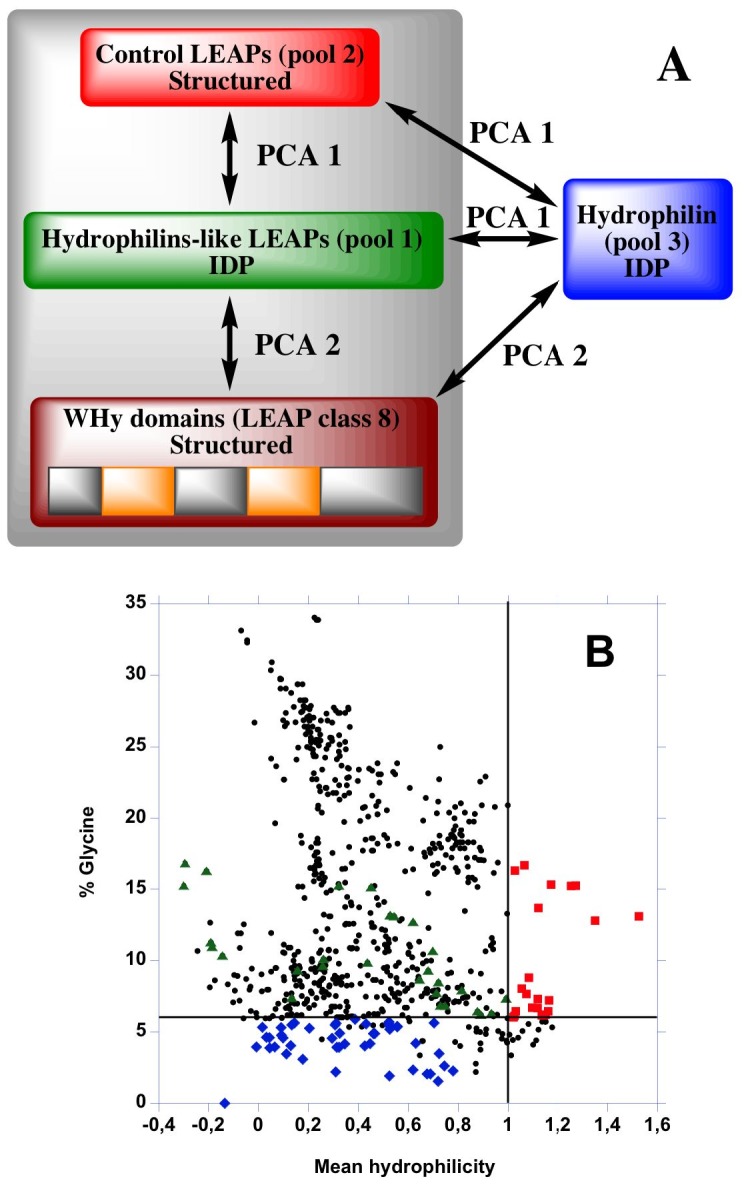
Schematic representation of the approach for the study of LEAPs, hydrophilins and [WHy domain/LEAP class 8]. (A) PCA 1: principal component analysis of the three pools. PCA 2: principal component analysis of [WHy domain/LEAP class 8] *vs*. [hydrophilins/LEAP class 2]. IDP: intrinsically disordered proteins. (B) Distribution of hydrophilins-like LEAPs, control LEAPs, hydrophylins and other LEAPs. Red squares: pool 1 (hydrophilins-like LEAPs) retrieved from LEAPdb characterized by %Gly>6%, GRAVY<−1 and mean hydrophilicity>1. Blue rectangles: pool 2 (control LEAPs) retrieved from LEAPdb characterized by %Gly<6%, GRAVY>−1 and mean hydrophilicity<1. Green triangles: pool 3 (hydrophilins) characterized by 6,2<%Gly<16,8%, −1,86<GRAVY<−1, −0,3<mean hydrophilicity<1. Black circles: all other LEAPs.

We performed a detailed comparison of LEAPs amino acid usage, amino acid physico-chemical properties with those of hydrophylins and WHy domain (Figure 1A). The overall analysis indicates that LEAPs are not a protein subset of hydrophilins family. Hydrophilins are rather related to LEAP class 2 (also called dehydrins) and to HSP12. It also suggests and/or confirms that LEAP class 2, hydrophilins and WHy domain interact with water or other polar/charged small molecules, and thus could share a common physiological role in dehydration tolerance.

## Methods

Many graphics shown in this study and many hundred other can be automatically generated online using the « *Statistical analysis* » option of the web interface of LEAPdb (http://forge.info.univ-angers.fr/~gh/Leadb/index.php).

### Boxplots

Each box encloses 50% of the data with the median value of the variable displayed as a line. The top and bottom of the box mark the limits of ±25% of the variable population. The lines extending from the top and bottom of each box mark the minimum and maximum values within the data set that fall within an acceptable range. Outliers points are points whose values are either greater than upper quartile + (1.5× interquartile distance) or less than lower quartile - (1.5× interquartile distance).

### Mean net charge *vs.* mean hydrophobicity and mean net charge *vs.* mean hydropathy plots

The mean net charge at pH 7 is the net charge of the polypeptide at pH 7 calculated using the pKa of the residues divided by the length of the sequence. The mean normalized net charge at pH 7.0 (<R>) is the mean net charge at pH 7.0 normalized between 0 and 1 [32]. GRAVY (grand average of hydropathy) is calculated by adding the hydropathy value of all residues divided by the number of residues in the polypeptide. The hydropathy scale used is that of Kyte and Doolittle [33]. The normalized GRAVY is the GRAVY normalized between 0 and 1 [32]. The mean hydrophobicity <H> is the sum of the hydrophobicity, using the hydrophobicity scale of Eisenberg *et al*. [34], of all residues divided by the number of residues in the polypeptide. The mean normalized hydrophobicity (normalized <H>) is the mean hydrophobicity normalized between 0 and 1.

### The 12 LEAP classes

Data about LEAPs contained in LEAPdb [8] were used. LEAPs have been rigorously classified into 12 non-overlapping classes. Each class contains various number of sequences characterized by: (i) a unique amino acid motif; (ii) a homogeneous PFAM [35], Interpro [36] and CDD [37] annotations. LEAPdb provides a large number of physico-chemical properties: number of amino acids (length), molecular weight, FoldIndex [38], isoelectric point (pI), mean (reduced) net charge at pH 7, mean hydrophilicity [39], GRAVY, mean hydrophobicity (<H>), mean bulkiness [40], mean average flexibility [41], mean molar fraction of accessible residues [42], mean molar fraction of buried residues [42], mean transmembrane tendency [43] and the percentage of each amino acid. From all those data, we calculated additional data such as fractional content of combinations of specific amino acids residues, and the relative usage of each amino acid by LEAPs compared to all known proteins (*i.e.*, the Uniprot release of 2013_03) [44]. The same types of data were calculated for hydrophilins and WHy domain and further compared to those of LEAPs.

### Hydrophilins and HSP12 dataset

Hydrophilins were initially characterized by a Gly content > 6%, GRAVY<−1 and a mean hydrophilicity>1 [27], [30]. To take in account the overlap with LEAPs, three pools of proteins were built (Figure 1B). Pool 1 - « hydrophilins-like LEAPs »: only 24 LEAPs are characterized by %Gly>6%, GRAVY<−1 and mean hydrophilicity>1 (pool 1). They belong to LEAP classes 1, 2, 3 and 5 (and correspond to 2, 14, 7 and 1 LEAPs, respectively). Pool 2 - « control LEAPs »: it contains 47 LEAPs with values opposite to those characterizing hydrophilins (*i.e.*, %Gly <6%, GRAVY>−1 and mean hydrophilicity<1). It contains LEAPs from LEAP classes 6, 7, 9 and 10 (24, 11, 11 and 1 LEAPs, respectively). It must be noticed that only one LEAP has no Gly (LEAP Class 7 - Acc#ACJ83952 from *Medicago truncatula*). Pool 3 - hydrophilins: Their sequences were retrieved from the public database NCBI, using hydrophilin-linked keywords and literature sources [27]-[30], [45], [46]. Blasting sequences previously obtained retrieved additional sequences. 159 sequences were thus obtained. Among them, 35 sequences were rejected because they have a %Gly<6% and/or a GRAVY>−1 and 86 sequences were rejected because they were redundant. It must be noticed that most of sequences are very poorly or even not annotated and that the hydrophilin-like superfamily clan (CL0385) includes PF00477 (*i.e.*, LEAP class 5 [8]). Finally, 31 sequences were retained as true hydrophilins. Sequences accession numbers of the three pools are listed in Table S1.

It has been shown that HSP12 from yeast is a hydrophilin [27]. HSP12 is also an IDP that modulates membrane function [47]. We have included HSP12 in our analysis as an additional dataset in order to compare it with LEAPs and hydrophilin.

### Sequences containing WHy domain

All LEAP class 8 contain a WHy domain (smart00769, CDD129008, IPR013990). The sequence of this domain was manually extracted from each sequence of LEAP class 8 using a PHP script.

### IDP dataset

Sequences corresponding to GRAS proteins (gibberellic acid insensitive (GAI), repressor of GAI, Scarecrow) were collected [48]. Plant IDPs were searched using DisProt [49] and « Entrez » (NCBI). We also searched archetypal IDP or IDR such as p53, abscisic stress ripening protein, CREB-binding protein, proteins related to DNA binding or processing, transcription regulation (cyclin-dependent kinase inhibitor, histone) and specific plants proteins (glutenin, Calvin cycle enzymes). Additional sequences were obtained by BLAST: only sequences having more than 50% identity with the query sequence were kept. Among the results, only fully annotated files corresponding to full-length sequences were retained. Finally, to ensure their IDP character, we retained only 72 sequences with FoldIndex≤0.

### FS dataset

A set of 158 fully structured proteins with known 3-D structures was selected from the PDB select 25 file: all proteins have less than 25% sequence identity with high quality X-ray crystallography resolution (<3.5 Angstroms).

### Data for the statistical analysis

We used three groups of properties for the sequences: a first group of 12 physico-chemical properties (set 1), a second group of 20 relative counts of amino acids (set 2), a third group of 11 combination of plain percentages of amino acids (set 3), thus leading to a total of 43 properties (Table S2).

### Methods for both statistical analyses (three pools and four sets)

After a first global non-parametric comparison (Kruskall-Wallis Rank Sum test), we first performed a classical one-way statistical analysis with descriptive computations, a comparative non-parametric test (Mann Whitney test) and a visual comparison (boxplots) for all the properties. We then realized 4 PCA (normed principal component analysis), one for each group of properties and a fourth using the 43 properties altogether. The last part of the analysis dealt with the extraction of the most contributing variables to the first factorial axis in order to build a table of most significant properties. Statistical significance was determined at the level p = 0.05. Non-parametric were preferred since normality was not clearly demonstrated and because of the small size of pool 1 and pool 3 (n = 24 and 31, respectively).

## Results

### Characteristics of hydrophilins and LEAPs datasets

The distribution of the three pools plus all remaining LEAPs from LEAPdb was plotted as a function of their %Gly and their mean hydrophilicity (Figure 1B). 622 LEAPs have %Gly>6% (with a maximum at 34,1%). LEAP pool with %Gly>6% and hydrophilicity>1 belong to class 1 and 2.

An interesting point is the diversity of organisms from which hydrophilins were retrieved (Table S1): 13 organisms are Fungi (*Ascomycota*; *Saccharomycetales*) and 1 organism is a nematode (*Caenorhabditis remanei*; *Metazoa*; *Nematoda*)].

### Characteristics of WHy domain and LEAP class 8 datasets

**Figure 2:**
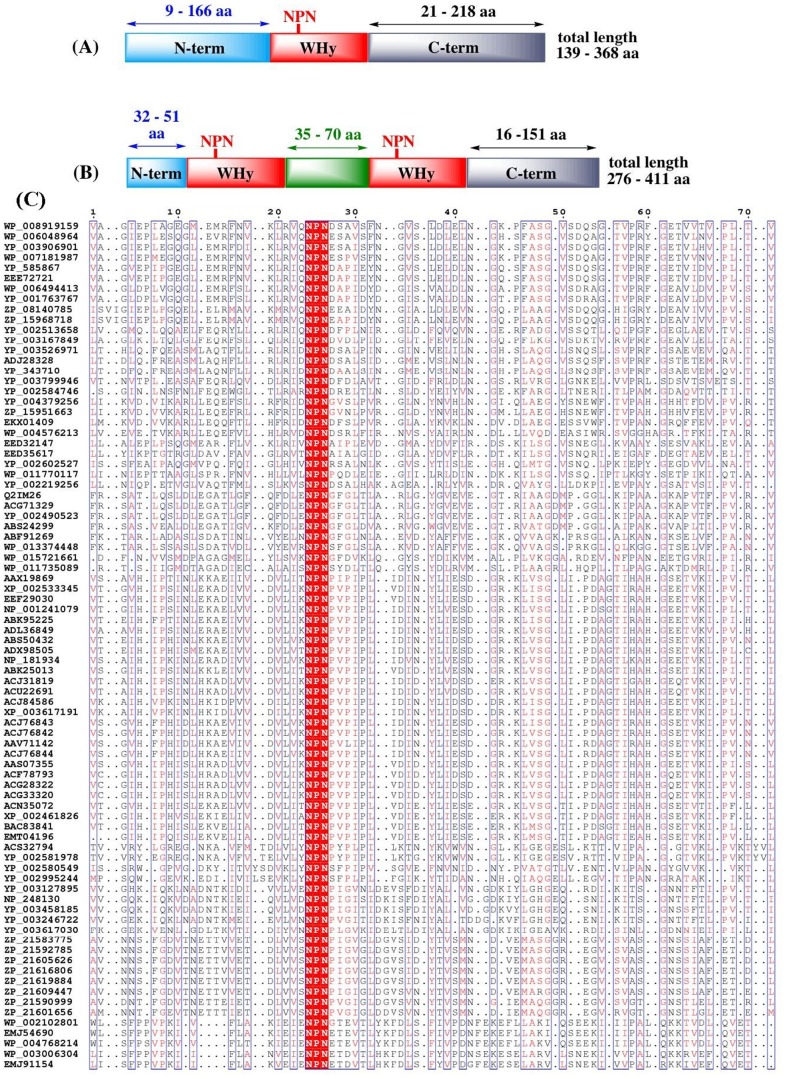
Schematic representation of WHy domain. (A) WHy domain contains an invariant triplet NPN situated 25 amino acids after the beginning of the WHy domain. (B) Some LEAP class 8 sequences contain a second WHy domain whose consensus sequence is very similar to the first domain. (C) Alignment of WHy domain sequences. The amino acids consensus sequence around the invariant triplet NPN can be written as: [ALMNV].{0,4}[FILMVWY].[AFILMV].{1,3}[FLMVY].[AILV].NPN.{3,3}[ILV].[AFILVY].{2,4}[FILMVY].{1,2}[FLVWY].[ILV].

146 LEAP class 8 contain one WHy domain and 16 LEAP class 8 contain an additional consensus sequence corresponding to the signature of this domain. The WHy domain can be described as following (Figure 2A): (i) it has a length of roughly 100 amino acids, beginning 9 to 166 amino acids from the N-terminal extremity (75% of the N-terminal domains have a length less or equal to 46 amino acids) and ending 21 to 218 amino acids from the C-terminal extremity (75% of the C-terminal domains have a length less or equal to 42 amino acids); (ii) it contains an invariant triplet NPN (NPL is found in only 3 sequences upon 159 LEAP class 8) situated 25 amino acids after the beginning of the WHy domain; (iii) it corresponds to a very conserved stretch of [aliphatic or hydrophobic or aromatic] residues separated by [charged or polar] ones; (iv) the amino acids consensus sequence around the invariant triplet NPN can be written as: [ALMNV].{0,4}[FILMVWY].[AFILMV].{1,3}[FLMVY].[AILV].NPN.{3,3}[ILV].[AFILVY].{2,4}[FILMVY].{1,2}[FLVWY].[ILV] with «.»  =  any amino acid, {n,m}  =  any amino acid n to m times, [XY] = X or Y; (v) the predicted secondary structure of the WHy domain corresponds to beta strands followed by a C-terminal alpha helix (not shown).

16 LEAP class 8 sequences contain a second WHy domain with an internal domain separating the two WHy domains whose length ranges from 35 to 70 amino acids (Figure 2B). The consensus sequence of the second WHy domain is very similar to the first one.

### Comparison of LEAPs, hydrophilins, WHy domain and HSP12 physico-chemical properties

Mean values are uniformly more predictive than total values for significantly correlated parameters [50]. LEAPs and hydrophilins have roughly the same values of pI, mean net charge at pH 7. This is logical since these physico-chemical properties are the criteria of initial selection. Hydrophilins-like LEAPs (pool 1) have a very high mean hydrophilicity. Control LEAPs (pool 2) have a lower mean hydrophilicity comparable to that of hydrophilins (pool 3).

**Figure 3:**
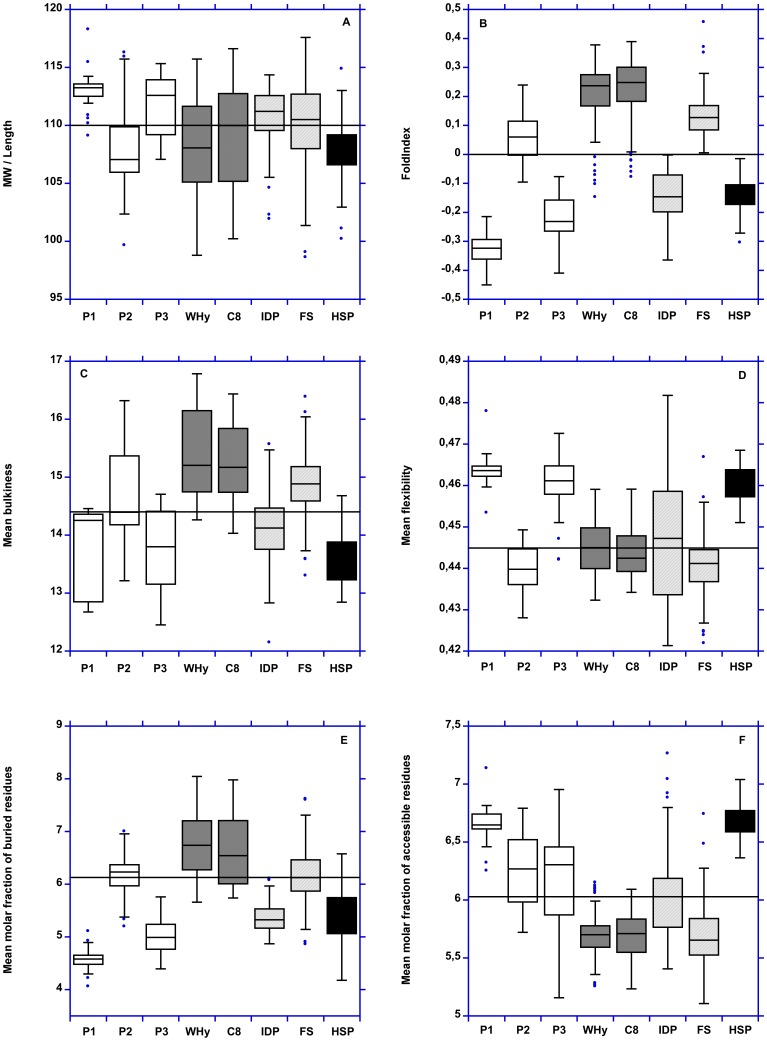
Boxplot representation of MW/length ratio, FoldIndex, mean bulkiness and mean flexibility, mean molar fraction of buried residues and mean molar fraction of accessible residues. P1: pool 1. P2: pool 2. P3: pool 3. C8: LEAP class 8. IDP: intrinsically disordered proteins. FS: fully structured proteins. HSP: HSP12.

**Figure 4:**
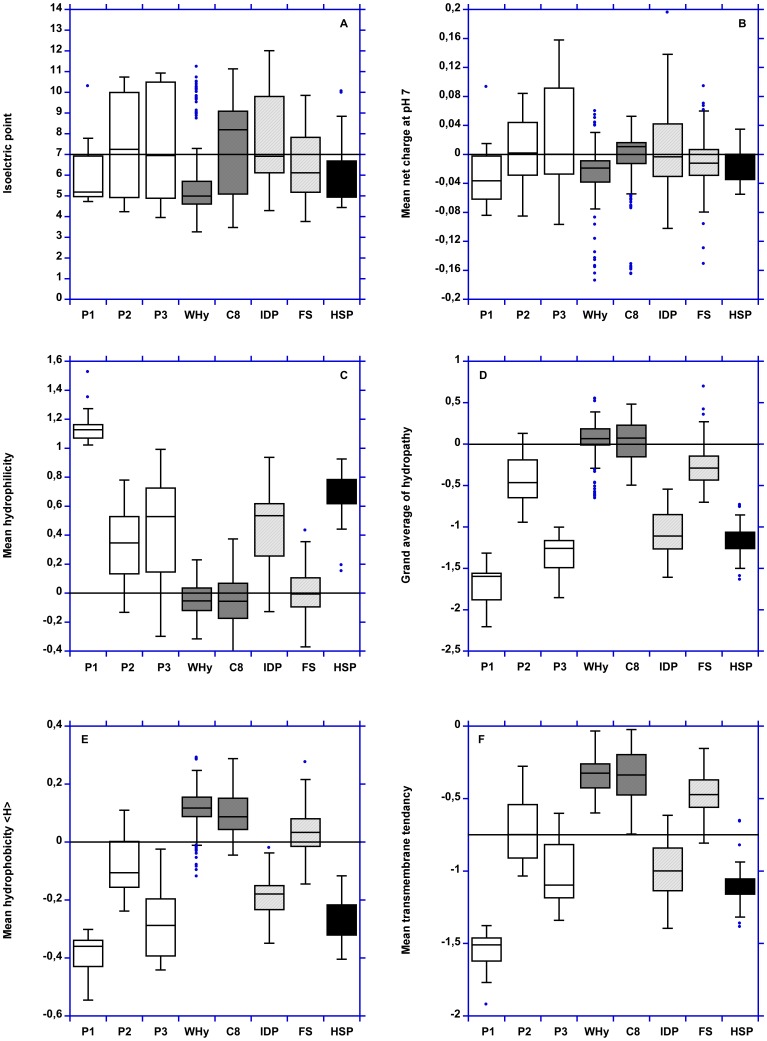
Boxplot representation of isoelectric point, mean net charge at pH 7, mean hydrophilicity, mean normalized GRAVY, mean normalized hydrophobicity (<H>) and mean transmembrane tendency. P1: pool 1. P2: pool 2. P3: pool 3. C8: LEAP class 8. IDP: intrinsically disordered proteins. FS: fully structured proteins. HSP: HSP12.

**Figure 5:**
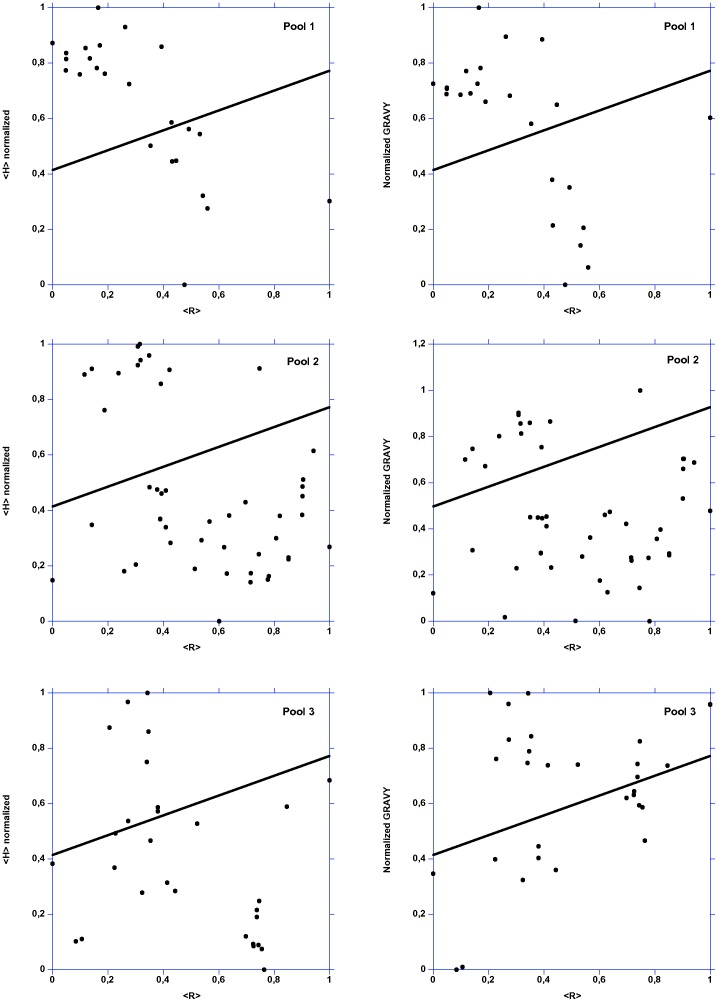
Mean normalized hydrophobicity (<H>) *vs.* mean net charge (<R>) plot and mean normalized GRAVY *vs.* mean net charge (<R>) plot for the three pools. The two areas are delimited by lines corresponding to the following equations, respectively: normalized <H> or normalized GRAVY = 0,359 <R>+0,413. The lines thus indicate the boundary between folded (above) and unfolded (below) polypeptide chains.

LEAPs and hydrophilins differ for the other physico-chemical properties, especially FoldIndex, mean bulkiness, mean flexibility, mean molar fraction of buried residues, mean transmembrane tendency and global hydrophobicity (GRAVY and <H>) (Figures 3 and 4). Conversely, for these two last properties, hydrophilins are closer to «hydrophilins-like LEAPs» (Figure 5).

Natively folded proteins and IDP occupy non-overlapping regions in the mean net charge *vs.* mean hydrophobicity plots, with natively IDP localized below a zone delimited by a line whose equation is: <H> normalized  = (<R>+1,151)/2,785 [32]. It has been shown that the combination of low mean hydrophobicity (*i.e.*, less driving force for protein compaction) and relatively high mean net charge (*i.e.*, charge - charge repulsion) is important for the absence of compact structure in proteins under physiological conditions [51].

Most of «control LEAPs» are localized below the line while most of «hydrophilins-like LEAPs» and hydrophilins are localized above that line (Figure 5), thus hydrophilins appear more natively folded than LEAPs. These results are confirmed by plotting the charge - hydropathy distribution, *i.e.*, normalized GRAVY *vs.* <R> normalized (Figure 5).

The comparison of the physico-chemical properties of the three pools leads to the conclusions that: (i) hydrophilins differ from LEAPs except LEAP class 2; (ii) a pertinent and precise definition of hydrophilins remains to be obtained (*i.e.*, %Gly> 6%, GRAVY <−1 and mean hydrophilicity> 1 is not sufficient); (iii) it is likely that «hydrophilins-like LEAPs» are «borderline» LEAPs. It must be noticed that 622 LEAPs have %Gly> 6% (increasing up to 34,1%). Moreover, LEAPs with %Gly> 6% and hydrophilicity> 1 belong to classes 1 and 2.

Hydrophilins-like LEAPs (pool 1) has identical (although more marked) physico-chemical properties as hydrophilins (pool 3) [PCA1, Figure 1A]. Among the three pools, pool 2 (control LEAPs) is the closest to WHy domain [PCA2, Figure 1A]. On the contrary hydrophilins have physico-chemical properties opposite to those of WHy domain [PCA2, Figure 1A]. WHy domain and LEAP class 8 have identical physico-chemical properties except for pI and mean net charge at pH7.

HSP12 and hydrophilins have identical physico-chemical properties although HSP12 are slightly more acidic (pI and mean net charge at pH 7 - Figure 4). This result confirms that HSP12 are related to hydrophilins [27].

All the physico-chemical properties described above were also expressed in a binary mode (Table 1), in order to reflect the distribution of each class with reference to the overall median or a reference value (e.g., 7 for pI). The values obtained for the 12 LEAP classes [26] have been added for a better comparison with hydrophilins, WHy domain and HSP12.

**Table 1:** Binary^a^ representation of the physico-chemical properties distribution of « hydrophilins-like LEAPs » (pool 1), « control LEAPs » (pool 2), hydrophilins (pool 3), HSP12, WHy domain and LEAP class 8.

Physico-chemical property	FoldIndex	Mean bulkiness	Mean flexibility	MBR^b^	MAR^c^	MTT^d^	pI	MNC pH 7^e^	MH^f^	GRAVY^g^	<H>^h^
Pool 1	−1	+1	+1	−1	+1	−1	−1	−1	+1	−1	−1
Pool 2	+1	+1	−1	+1	+1	+1	+1	+1	+1	−1	−1
Pool 3	−1	−1	+1	−1	+1	−1	+1	0	+1	−1	−1
HSP12	−1	−1	+1	−1	+1	−1	−1	−1	+1	−1	−1
WHy domain	+1	+1	+1	+1	−1	+1	−1	−1	−1	+1	+1
LEAP class 8	+1	+1	−1	+1	−1	+1	+1	+1	−1	+1	+1
IDP^i^	−1	+1	−1	−1	−1	−1	−1	−1	+1	−1	−1
FS^j^	+1	+1	−1	+1	−1	+1	−1	−1	−1	−1	+1

These data are compared to two control datasets (IDP and FS datasets).

a Values +1, 0 and −1 values mean that the physico-chemical properties considered is upper, equal or lower, respectively, than either the calculated median value for the seven datasets or a definite « natural » value (see the corresponding figures).

b Mean molar fraction of buried residues.

c Mean molar fraction of accessible residues.

d Mean transmembrane tendency.

e Mean net charge at pH 7.

f Mean hydrophilicity.

g Grand average of hydropathy.

h Mean hydrophobicity.

i Intrinsically Disordered Proteins dataset.

j Fully Structured proteins dataset.

### Comparison of LEAPs, hydrophilins, WHy domain and HSP12 amino acids usage

#### Percentage of amino acids

Surprisingly, the Gly content (Figure S1A) of hydrophilins is not so important: up to 16,8%, *i.e.*, much less than the 34,1% for LEAP class 1 (PF00257). Hydrophilins have the highest content in Asn and Gln (Figures S1B & S1C). Glu is largely more used than Asp in the case of «hydrophilins-like LEAPs» and in the same manner in those of true LEAPs and hydrophilins (Figures S1D & S1E). «Hydrophilins-like LEAPs» have the highest content of Glu and Lys leading to an acidic pI. Lys is largely more used than Arg in the case of «hydrophilins-like LEAPs» and to a less extent in that of true LEAPs (pool 2) (Figures S1F & S1G). True LEAPs have a very high content in Ala (Figure S1H) and may be linked to the GRAVY and <H> values observed for true LEAPs (Figure 5). The three pools have no or very low content of Cys and Trp (Figures S2C & S2E). It is thus unlikely that hydrophilins contains disulfide bridges.

#### Order and disorder promoting residues

**Figure 6:**
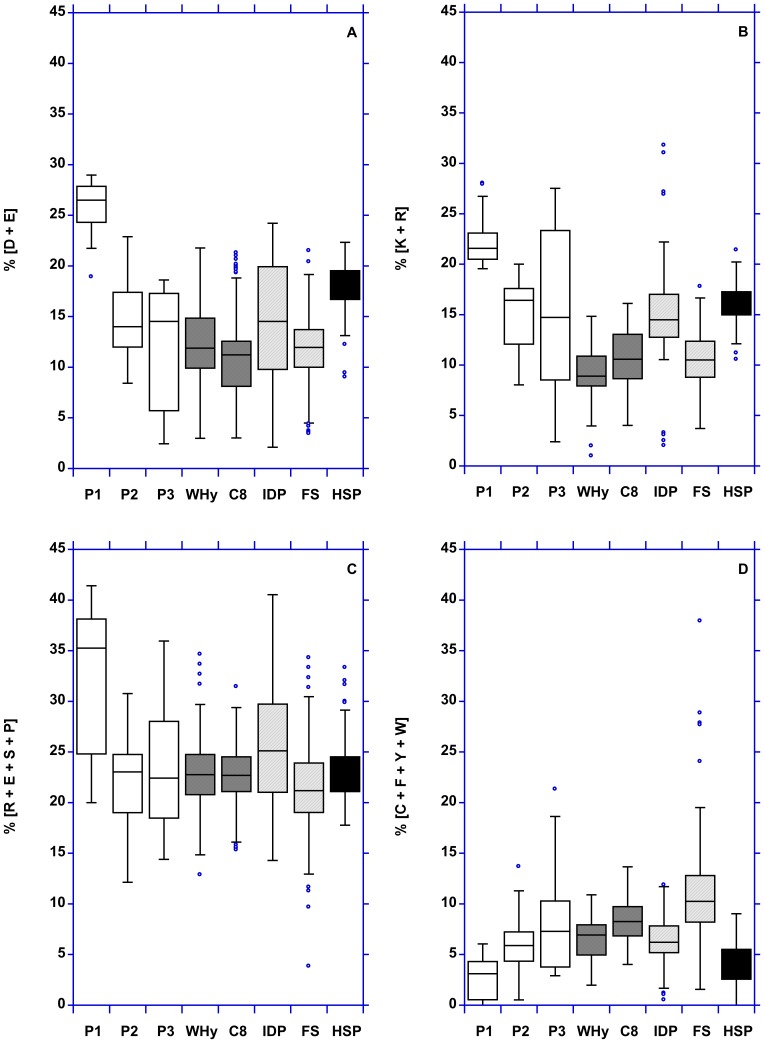
Fractional content (*i.e.*, the sum of residues normalized by protein chain-length) of some particular amino acids combinations. (A) Negatively charged residues (Asp + Glu). (B) Positively charged residues (Arg + Lys). (C) Strongest disorder promoting residues (Arg + Glu + Ser + Pro). (D) Strongest order promoting residues (Cys + Phe + Tyr + Trp). P1: pool 1. P2: pool 2. P3: pool 3. C8: LEAP class 8. IDP: intrinsically disordered proteins. FS: fully structured proteins. HSP: HSP12.

The use of Asp and Glu can be represented also as the fractional content of negatively charged residues [50] *i.e.*, the number of Asp plus Glu residues, normalized by protein chain-length (Figure 6A). The use of Arg and Lys can be also represented as the fractional content of positively charged residues [50] *i.e.*, the number of Arg plus Lys residues, normalized by protein chain-length (Figure 6B). Pool 1 has the highest [R+E+S+P/length] ratio, (*i.e.*, the strongest disorder promoting residues [52]) and the lowest [C+F+Y+W/length] ratio (*i.e.*, the strongest order promoting residues) (Figures 6C & 6D). However, there is no net difference between hydrophilins and WHy domain since the range of values for hydrophilins (box-plots) is very large. Nevertheless, this result suggests that WHy domain is structured. The results for HSP12 are comparable to those for hydrophilins. It must be noticed that only 2 and 6 HSP12 sequences (upon 60) contain Cys and Trp, respectively.

#### Frequency of usage of each amino acid

The percentage of each amino acid was calculated for each of the three pools and WHy domain. This value was then divided by the percentage of each amino acid found in release 2013_03 of UniProtKB/Swiss-Prot. This ratio thus describes the frequency of usage of each amino acid (Figures S3 & S4). In other words, a value of 1 means the usage of a given amino acid is the same as its usage by all proteins contained in Uniprot (Table 2). Pool 1 is characterized by a high level of Glu, Lys and especially His and a depletion of Asn, Gln, Arg, hydrophobic residues, aromatic residues, Cys, Thr and Met. Pool 3 is characterized by a high level of Gly, Asn, Gln, Lys and Tyr and a depletion of hydrophobic residues, Phe, Trp and Cys. WHy domain is characterized by a high level of Asn, Val and Pro and a depletion of Cys, Met and His.

**Table 2:** Binary^a^ representation of amino acids usage by « hydrophilins-like LEAPs » (pool 1), « control LEAPs » (pool 2), hydrophilins (pool 3), LEAP class 8 and WHy domain compared to the overall proteins contained in Uniprot.

Amino acid	A	C	D	E	F	G	H	I	K	L	M	N	P	Q	R	S	T	V	W	Y
Pool 1	−1	−1	−1	+1	−1	+1	+1	−1	+1	−1	−1	−1	+1	−1	−1	+1	−1	−1	−1	−1
Pool 2	+1	−1	+1	+1	−1	−1	−1	−1	+1	−1	−1	−1	−1	−1	−1	+1	−1	+1	−1	−1
Pool 3	−1	−1	−1	−1	−1	+1	−1	−1	+1	−1	−1	+1	−1	+1	+1	+1	−1	−1	−1	+1
WHy domain	−1	−1	−1	−1	−1	+1	−1	−1	−1	+1	−1	+1	+1	−1	−1	−1	−1	+1	−1	−1
LEAP class 8	−1	−1	−1	−1	+1	+1	−1	+1	−1	+1	−1	−1	+1	−1	−1	−1	−1	+1	−1	−1
IDP^b^	−1	−1	−1	+1	−1	+1	+1	−1	+1	−1	−1	−1	+1	−1	−1	−1	−1	−1	−1	−1
FS^c^	−1	−1	+1	−1	−1	+1	+1	−1	−1	−1	−1	+1	−1	−1	−1	−1	+1	+1	+1	+1

These data are compared to two control datasets (IDP and FS datasets).

a Values +1 and −1 indicate that the median value of the ratio (% amino acid considered in LEAP/% amino acid considered in Uniprot) is upper or lower than 1 (see the corresponding figures).

b Intrinsically Disordered Proteins dataset.

c Fully Structured proteins dataset.

### Principal component analysis (PCA)

#### Analysis of the three pools and HSP12

**Figure 7:**
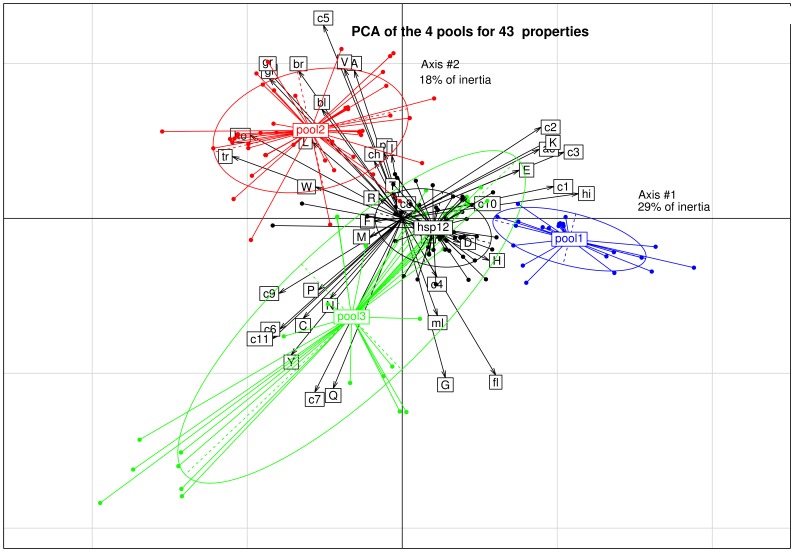
Principal component analysis of the three pools and HSP12. Abbreviations used: **(i) Physicochemical properties**: isoelectric point: pi, FoldIndex: gi, GRAVY: gr, net charge at pH 7: ch, mean hydrophilicity: hi, hydrophobicity (<H>): ho, mean average flexibility: fl, mean bulkiness: bl, mean molar fraction of buried residues: br, mean molar fraction of accessible residues: ac, mean transmembrane tendency: tr, molecular weight/length: ml. **(ii) Ratio [(%amino acid X)/(% amino acid X Uniprot)]**: pctA.Unip: A, pctC.Unip: C, pctD.Unip: D, pctE.Unip: E, pctF.Unip: F, pctG.Unip: G, pctH.Unip: H, pctI.Unip: I, pctK.Unip: K, pctL.Unip: L, pctM.Unip: M, pctN.Unip: N, pctP.Unip: P, pctQ.Unip: Q, pctR.Unip: R, pctS.Unip: S, pctT.Unip: T, pctV.Unip: V, pctW.Unip: W, pctY.Unip: Y. **(iii) Amino acids combination**: [D+E]: c1, [K+R]: c2, [D+E+K+R]: c3, [D+E−K−R]: c4, [A+I+L+V]: c5, [F+W+Y]: c6, [N+Q]:c7, [S+T]: c8, [C+W]: c9, [R+E+S+P]: c10, [C+F+W+Y]: c11.

Pool 1 and pool 3 are close, and pool 2 is clearly separated. HSP12 can be considered as included in pool 3 (Figure 7). This is best seen on the first of the four PCA that were analyzed, though it is not possible to prove it on the sole basis of the statistical tests, whether parametric or not (Table 3). The full PCA, with 43 properties, accounts for 68% of inertia on the first 4 axes, with already 47% of inertia on the first two axes (with respectively 29% and 18% of inertia).

**Table 3:** Normed principal component analysis (PCA) of the three pools plus HSP12.

Pools	Set 1 (12 properties)	Set 2 (20 properties)	Set 3 (11 properties)	Total (43 properties)
1 *vs*. 2	11	16	11	38
1 *vs*. 3	8	12	9	29
2 *vs*. 3	8	11	4	23
1 *vs*. [3 + HSP12]	9	13	10	32
2 *vs*. [3 + HSP12]	10	13	6	29

Median values for the most significant physico-chemical properties on the first two factorial axes of the PCA (Figure 7). The asterisk indicates the second axis.						
	HYDROPHI^a^	TRANSM	[D+E+K+R]	*[A+I+L+V]	*[N+Q]	*Q
Pool 1	1.12865	−1.50985	48.15252	16.00000	2.01051	0.33927
Pool 2	0.34690	−0.74830	28.43137	33.98693	5.76923	0.82303
Pool 3	0.52810	−1.09600	27.54491	14.21569	16.45570	1.38075
HSP12	0.70340	−1.12275	34.73857	20.09174	9.19540	1.44576

The number in each cell indicates the number of properties that are significantly different (p-value <0.05) using the non-parametric Mann Whitney test.

a HYDROPHI: mean hydrophilicity; TRANSM: mean transmembrane tendency.

#### Analysis of LEAP class 2, hydrophilins, HSP12, LEAP class 8 and WHy domain

**Figure 8:**
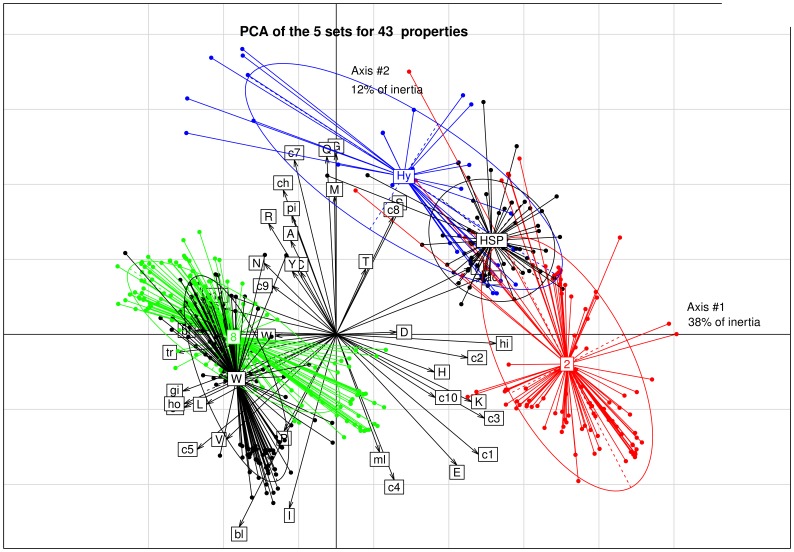
Principal component analysis of LEAP class 2, hydrophilins, HSP12, WHy domain and LEAP class 8. Abbreviations used: Hy: hydrophilins; W: WHy domain; 2: LEAP class 2; 8: LEAP class 8; HSP: HSP12. (i) Physicochemical properties: isoelectric point: pi, FoldIndex: gi, GRAVY: gr, net charge at pH 7: ch, mean hydrophilicity: hi, hydrophobicity (<H>): ho, mean average flexibility: fl, mean bulkiness: bl, mean molar fraction of buried residues: br, mean molar fraction of accessible residues: ac, mean transmembrane tendency: tr, molecular weight/length: ml. (ii) Ratio [(%amino acid X)/(% amino acid X Uniprot)]: pctA.Unip: A, pctC.Unip: C, pctD.Unip: D, pctE.Unip: E, pctF.Unip: F, pctG.Unip: G, pctH.Unip: H, pctI.Unip: I, pctK.Unip: K, pctL.Unip: L, pctM.Unip: M, pctN.Unip: N, pctP.Unip: P, pctQ.Unip: Q, pctR.Unip: R, pctS.Unip: S, pctT.Unip: T, pctV.Unip: V, pctW.Unip: W, pctY.Unip: Y. (iii) Amino acids combination: [D+E]: c1, [K+R]: c2, [D+E+K+R]: c3, [D+E−K−R]: c4, [A+I+L+V]: c5, [F+W+Y]: c6, [N+Q]:c7, [S+T]: c8, [C+W]: c9, [R+E+S+P]: c10, [C+F+W+Y]: c11.

Hydrophilins nearly includes HSP12 and is close to LEAP class 2. All these three sets of proteins are clearly apart from LEAP class 8 and WHy domain which are close (Figure 8). This is also best seen on the first PCA and moreover, the results of the statistical tests assert it (Table 4). The full PCA accounts for 67% of inertia for the first four axes, with main plane of axis 1 and axis 2 showing 50% of inertia (38% and 12% for axis 1 and axis 2, respectively).

**Table 4:** Normed principal component analysis (PCA) of LEAP class 2, hydrophilins, HSP12, LEAP class 8 and WHy domain.

	Set 1 (12 properties)	Set 2 (20 properties)	Set 3 (11 properties)	Total (43 properties)
Hydrophilins *vs*. LEAP class 2	7	14	10	31
Hydrophilins *vs*. LEAP class 8	10	15	5	30
Hydrophilins *vs*. WHy domain	12	15	7	34
Hydrophilins *vs*. HSP12	8	12	9	29
LEAP class 2 *vs*. LEAP class 8	12	17	10	39
LEAP class 2 *vs*. WHy domain	12	18	10	40
LEAP class 2 *vs*. HSP12	10	15	11	36
LEAP class 8 *vs*. WHy domain	4	13	9	26
LEAP class 8 *vs*. HSP12	12	17	10	39
WHy domain *vs*. HSP12	11	17	8	36

Median values for the most significant physico-chemical properties on the first two factorial axes of the PCA (Figure 8). The asterisk indicates the second axis.								
	HYDROPHI^a^	TRANSM	FI	GRAVY	HYDROPHO	*BULKI	*G	*Q
Hydrophylins	0.52810	−1.0960	−0.2316	−1.25980	−0.2875	13.8010	1.356190	1.380750
LEAP class 2	0.93960	−1.3740	−0.2721	−1.50960	−0.3190	14.1918	1.050910	0.601070
LEAP class 8	−0.05565	−0.3371	0.2480	0.07255	0.0875	15.1715	1.234015	0.895505
WHy domain	−0.07330	−0.3244	0.2410	0.07230	0.1150	15.7750	0.977530	1.007740
HSP12	0.70340	−1.12275	−0.15205	−1.18555	−0.25495	13.5468	1.267810	1.445760

The number in each cell indicates the number of properties that are significantly different (p-value <0.05) using the non-parametric Mann Whitney test.

a HYDROPHI: mean hydrophilicity; TRANSM: mean transmembrane tendency; FI: FoldIndex; GRAVY: grand average of hydropathy; HYDROPHO: mean hydrophobicity (<H>); BULKI: mean bulkiness.

IDP dataset and FS dataset were added to perform supplementary PCA (not shown). PCA of physicochemical properties (especially the FoldIndex parameter) confirms that hydrophilins are IDP, even though it is less obvious with PCA of amino acids.

## Discussion

WHy domain is characterized by the highest level of mean molar fraction of buried residues and the lowest level of mean molar fraction of accessible residues. This domain is likely compact with small cavities, if any, that can accommodate only small molecules. One of the best-documented LEAP's functions is their interaction with water and some polar cellular compounds [30]. Moreover, all LEAP classes (with exception of classes 7 and 8) are IDP [26]. This structural characteristic allows them to sequester water and sugars in a tightly hydrogen-bonded network [53], [54]. Thus, one of their noticeable physical properties is their ability to establish hydrogen bonds. The physico-chemical complexity of protein surfaces alters the structure of the surrounding layer of hydrating water molecules: hydration waters have slower correlation times than water in bulk [55]. Hydrogen bonds are established by area composed mainly by polar or polarizable amino acids such as Asn, Gln and Gly. The resulting area interacts more easily with polar molecules, especially water. WHy domain is composed of alternating hydrophobic and hydrophilic residues with an invariant NPN motif near its N-terminal extremity. A similar signature (NPA) linked to a crucial role in water transport is found in aquaporin [56]. It is possible that hydrophobic pockets create a barrier orienting the water molecule's dipole moment near the NPN motif.

Interactions between amino acids side chains and waters contribute to the stabilization of the native, thus functional, protein conformation. The interactions between water molecules and a small hydrophobic pentapeptide ([Ala]_5_), have been studied at controlled levels of hydration, by adding successively, up to 25 water molecules per peptide (this level corresponding to full hydration) [57]. The first added water molecules form naturally bonds with the hydrophilic part of the pentapeptide while the next added ones are confined to the surface of alanine without bond formation.

Plants exhibit a surveillance system based on disease resistance gene to recognize avirulence factors displayed by pathogens. Among defense responses activated after pathogen recognition, one is called hypersensitive response [58]. Some proteins (NDR1/HIN1-like [59] or harpin-induced-like gene 1 [60]) are coded NHL genes. WHy domain links NHL proteins to the plant family LEA-14. A link exists also between LEAPs class 6 (*i.e.*, group 3 cotton D-7 LEAP and group 3 cotton D-29 LEAP) [61]. Thus, it is likely that WHy domain play an important physiological role against pathogens-induced stress.

A protective role of hydrophilins against enzyme inactivation due to water limitation has been demonstrated [28]. They act as membrane and protein stabilizers during water stress, either by direct interaction or by acting as a molecular shield. It has been also shown that yeast Sip18 hydrophilin and STF2p hydrophilin from *Saccharomyces cerevisiae* have an antioxidative capacity under dehydration stress [29], [62].

The ratio [(%N+Q)/(%N+Q Uniprot)] and the ratio [(%A+I+L+V)/(%A+I+L+V Uniprot)] for hydrophilins are much higher and lower, respectively, than those of WHy domain/LEAP class 8: the overall polar character of hydrophilins is greater (Figures 7 & 8). PCA also clearly indicates that LEAP class 2 and hydrophilins have similar physicochemical properties and that LEAP class 8 and WHy domain have also similar physicochemical properties (Figure 8). In particular, the transmembrane tendency of hydrophilins (and LEAP class 2) is much lower than that of WHy domain (and LEAP class 8) indicating a greater propency of WHy domain to interact with membranes due probably to a stronger alpha helix dipolar moment. In addition, bulkiness of fully structured WHy domain is more pronounced than that of intrinsically disordered hydrophilins. It was shown the larger the hydrodynamic radius of the dehydrins (*i.e.*, LEAP class 2), the more effective their cryoprotant effect. LEAP class 2 and hydrophilins function as molecular shields, and their intrinsic disorder is required to be effective as cryoprotectant [63]. LEAPs, hydrophilins and WHy domain protect membranes against dehydration, but their protective action differ. LEAPs intrinsic disorder may provide hydrophilic surfaces ordering water molecules around proteins that stabilize these proteins [64]. Hydrophilins act as molecular shields *via* their intrinsic structural flexibility and prevent protein structure modification that is affected when water molecules are removed in the absence of a hydrophilin [64]. It was also proposed that hydrophilins mediate interactions with their target proteins or stabilize active conformation of enzymes [28]. Since recent studies provided no evidence for a membrane protective function of three LEAPs from class 8 [65], it can be hypothesized that WHy domain protects against water deficit rather through stabilization of membrane-bound proteins.

The assumption of Battaglia *et al*. [30] was based on few LEAPs sequences. This works provide new insights in LEAPs family: hydrophilins (at least those tested in this study) are likely a subset of the LEAPs family and belong to LEAP class 2 [8] also called dehydrins.

## Supporting Information

Figure S1 **Boxplot representation of amino acids percentages.** P1: pool 1. P2: pool 2. P3: pool 3. IDP: intrinsically disordered proteins. FS: fully structured proteins. Figures A to J: Gly, Asn, Glu, Asp, Gln, Lys, Arg, Ala, Ile, Leu, respectively.
(TIF)

Figure S2 **Boxplot representation of amino acids percentages.** P1: pool 1. P2: pool 2. P3: pool 3. IDP: intrinsically disordered proteins. FS: fully structured proteins. Figures A to J: Val, Phe, Trp, Tyr, Cys, Ser, Thr, Met, Pro, His, respectively.
(TIF)

Figure S3 **Boxplot representation of amino acids usage by the three pools compared to that of all proteins contained in Uniprot.** P1: pool 1. P2: pool 2. P3: pool 3. IDP: intrinsically disordered proteins. FS: fully structured proteins. Figures A to J: Gly, Asn, Gln, Asp, Glu, Lys, Arg, Ala, Ile, Leu, respectively.
(TIF)

Figure S4 **Boxplot representation of amino acids usage by the three pools compared to that of all proteins contained in Uniprot.** P1: pool 1. P2: pool 2. P3: pool 3. IDP: intrinsically disordered proteins. FS: fully structured proteins. Figures A to J: Val, Phe, Trp, Tyr, Cys, Ser, Thr, Met, Pro, His, respectively.
(TIF)

Table S1 (DOC)

Table S2 (DOC)
