# Supplementary figures and images for: Comparison of Amino Acids Physico-Chemical Properties and Usage of Late Embryogenesis Abundant Proteins, Hydrophilins and WHy Domain
Source: PLoS One. 2014 Oct 8;9(10):e109570. doi: 10.1371/journal.pone.0109570 (PMC4190154; doi:10.1371/journal.pone.0109570)

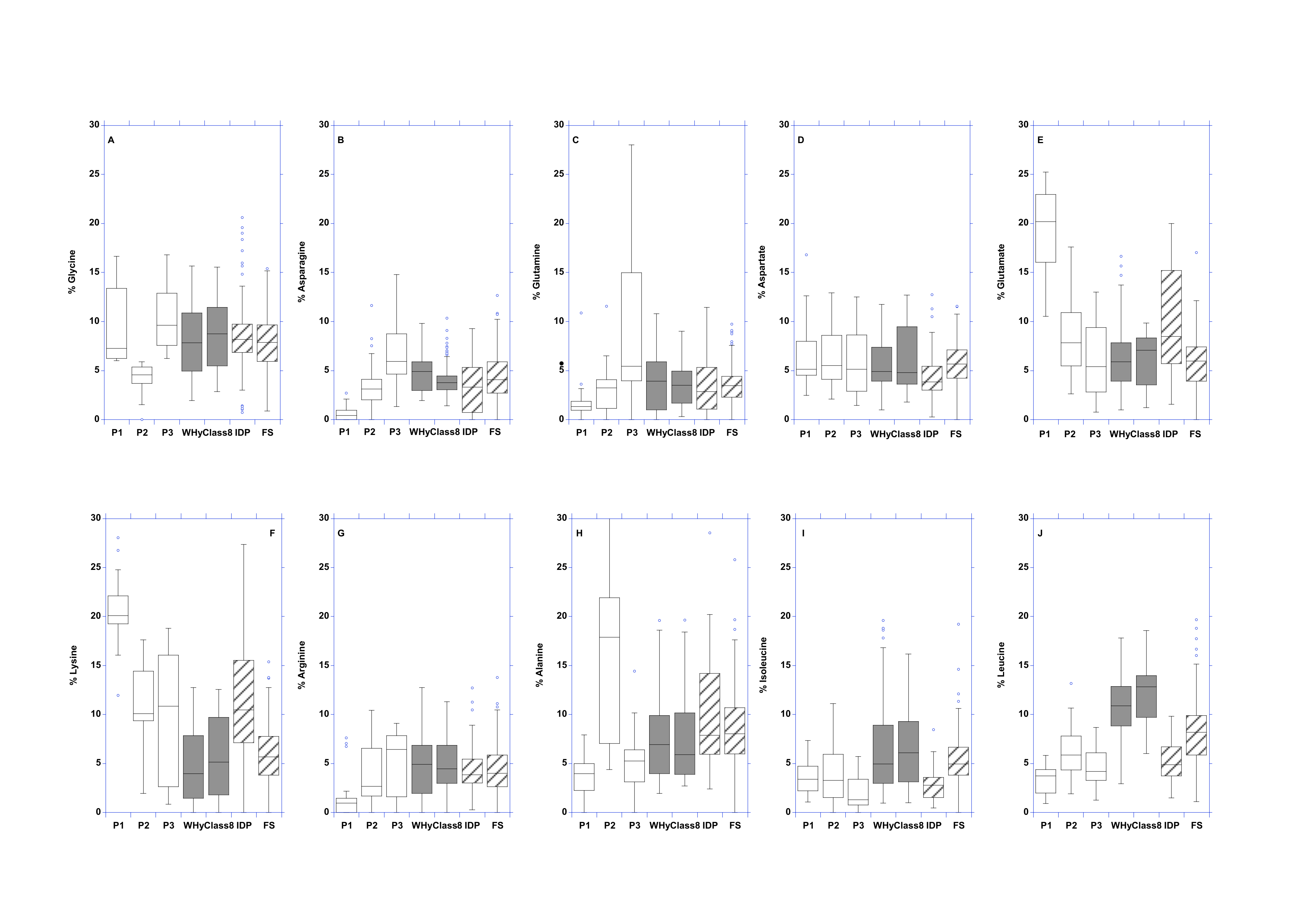

Supplement: Figure S1 — Boxplot representation of amino acids percentages. P1: pool 1. P2: pool 2. P3: pool 3. IDP: intrinsically disordered proteins. FS: fully structured proteins. Figures A to J: Gly, Asn, Glu, Asp, Gln, Lys, Arg, Ala, Ile, Leu, respectively. (TIF) [file pone.0109570.s001.tif]

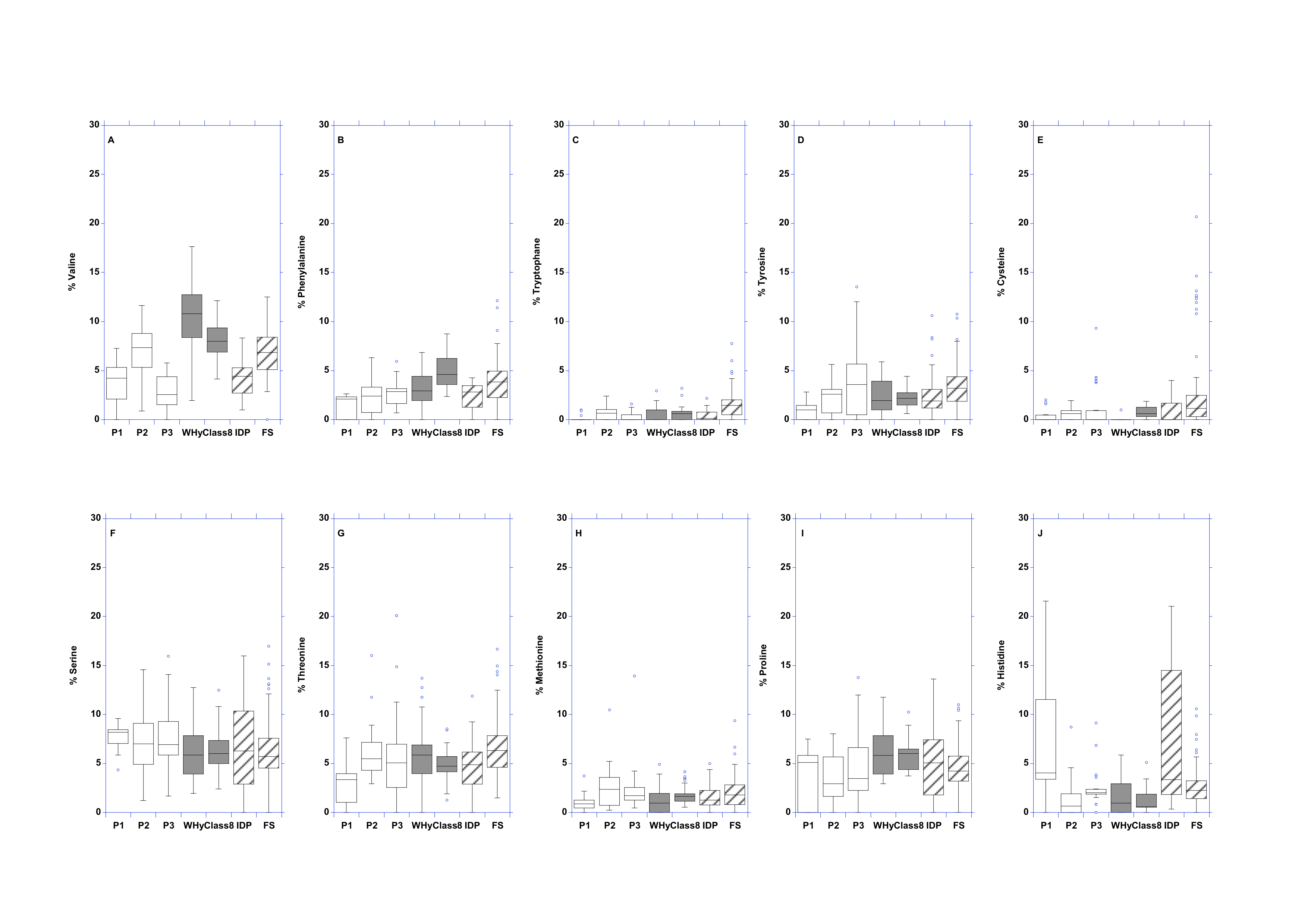

Supplement: Figure S2 — Boxplot representation of amino acids percentages. P1: pool 1. P2: pool 2. P3: pool 3. IDP: intrinsically disordered proteins. FS: fully structured proteins. Figures A to J: Val, Phe, Trp, Tyr, Cys, Ser, Thr, Met, Pro, His, respectively. (TIF) [file pone.0109570.s002.tif]

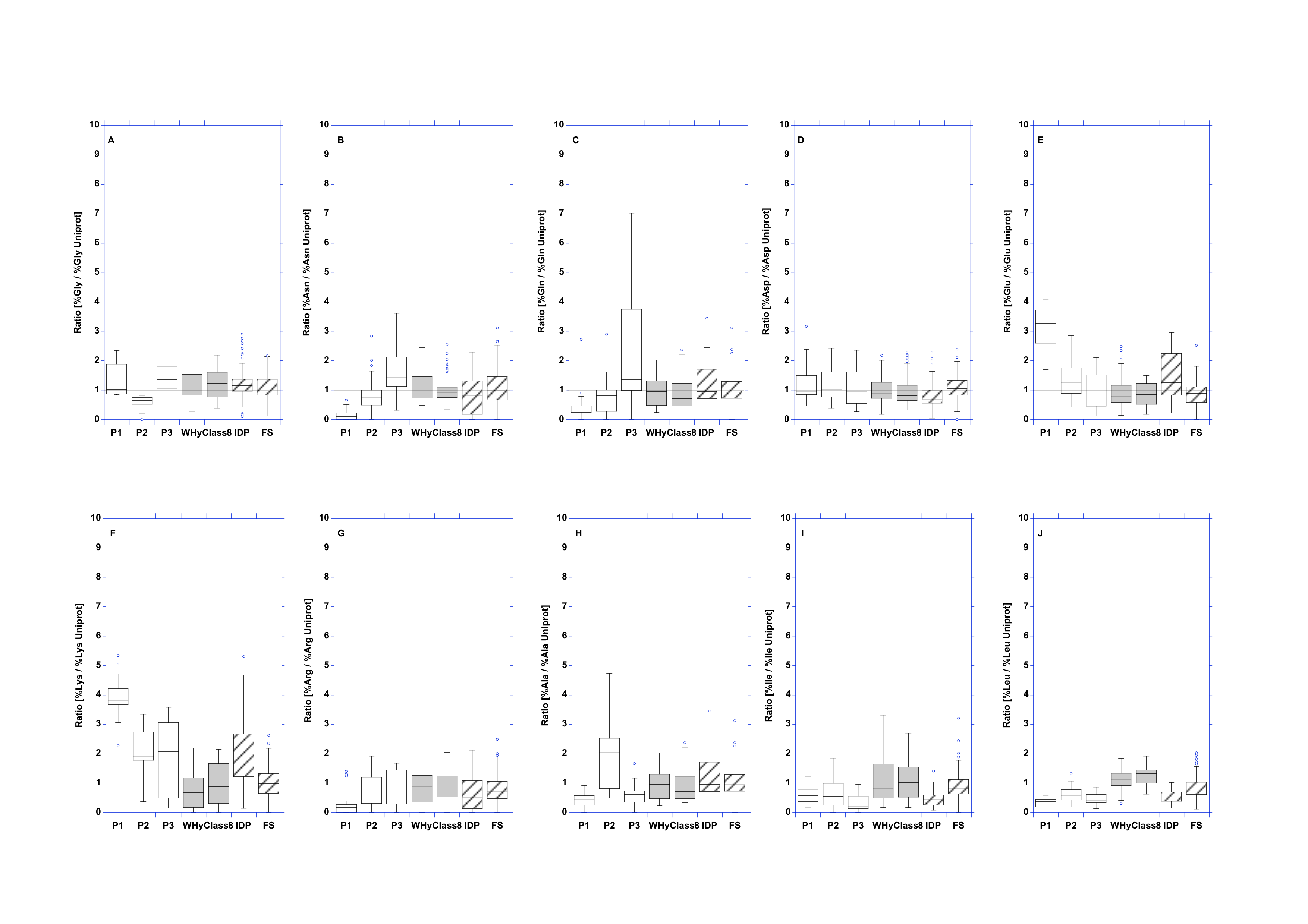

Supplement: Figure S3 — Boxplot representation of amino acids usage by the three pools compared to that of all proteins contained in Uniprot. P1: pool 1. P2: pool 2. P3: pool 3. IDP: intrinsically disordered proteins. FS: fully structured proteins. Figures A to J: Gly, Asn, Gln, Asp, Glu, Lys, Arg, Ala, Ile, Leu, respectively. (TIF) [file pone.0109570.s003.tif]

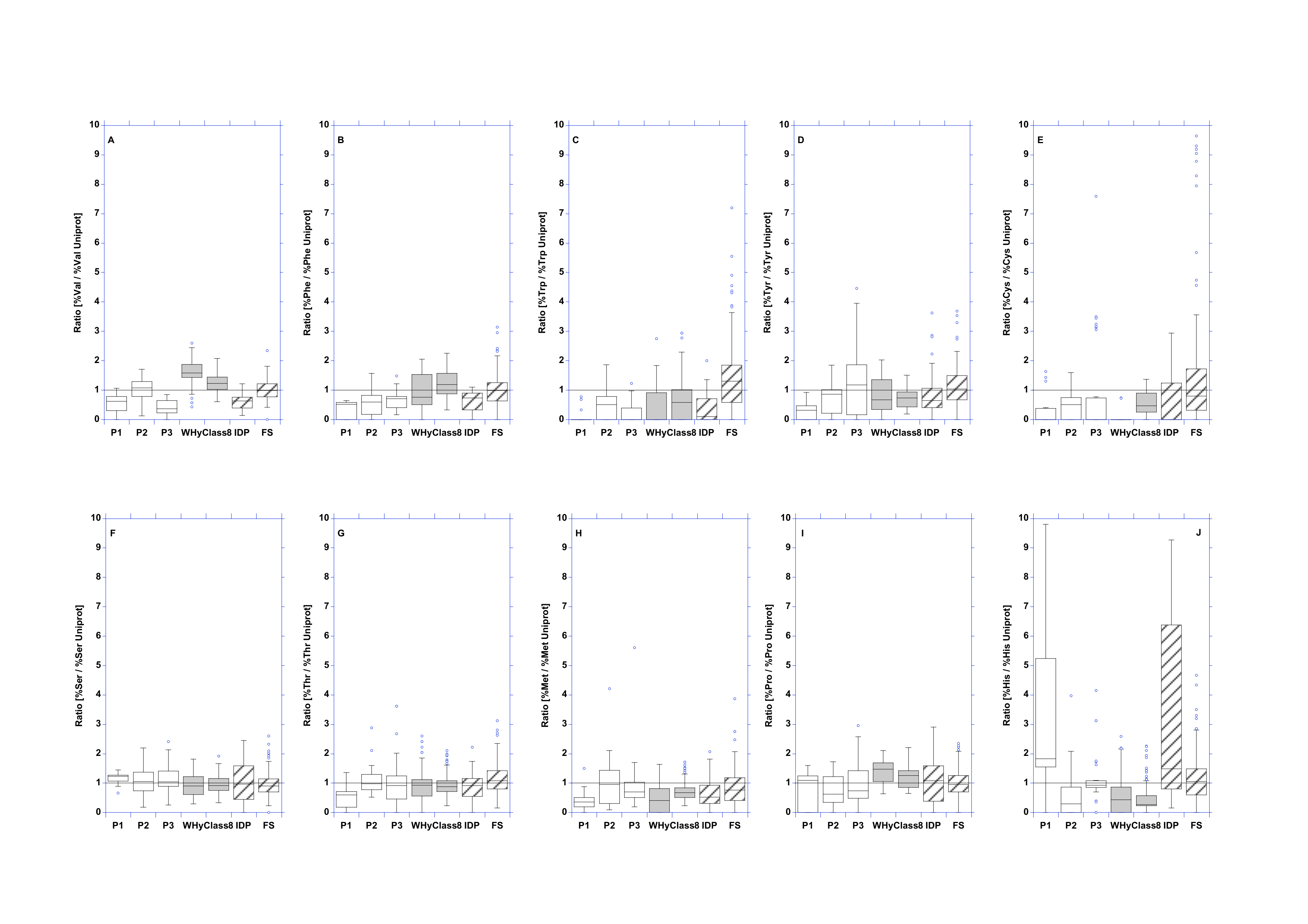

Supplement: Figure S4 — Boxplot representation of amino acids usage by the three pools compared to that of all proteins contained in Uniprot. P1: pool 1. P2: pool 2. P3: pool 3. IDP: intrinsically disordered proteins. FS: fully structured proteins. Figures A to J: Val, Phe, Trp, Tyr, Cys, Ser, Thr, Met, Pro, His, respectively. (TIF) [file pone.0109570.s004.tif]
