# Supplementary material for: Comparison of Amino Acids Physico-Chemical Properties and Usage of Late Embryogenesis Abundant Proteins, Hydrophilins and WHy Domain
Source: PLoS One. 2014 Oct 8;9(10):e109570. doi: 10.1371/journal.pone.0109570 (PMC4190154; doi:10.1371/journal.pone.0109570)
Supplement: Table S1 — (DOC) [file pone.0109570.s005.doc]

**Table S1.** Accession numbers of the proteins of the three pools.

| Pool 1 - « hydrophilins-like LEAPs » | | Pool 2 - « control LEAPs » | |
| --- | --- | --- | --- |
| AAK66763  ABF48481  ABH11546  ABS12333  ABS12334  ABS12347  ABS12348  AAB00554  AAU29458  AAB53203  AAP44575  BAD13499 | AAO38853  CAB93666  AAZ83586  ABC68275  AAN37899  AAT81473  AAS77419  AAT78838  AAX20043  NP_175843  CAA11834  BAA19768 | ACJ04786  ACJ86018  ACJ83952  AAD25354  P46519  AAL66195  CAA10047  EEF50596  ABC46705  AAB96796  NP_001147291  XP_002457757  AAD53078  EEF39286  YP_002701455  NP_001152662  ACV50424  NP_001148295  EEF29439  ABZ74500  ABU62808  ABU62809  ABU62811  ABU62810 | ABD57466  P71378  ABO84795  ABG54481  EEQ32719  ABD80469  ADD91479  ADD91471  EFE30227  EFE44586  BAE92616  ABM37820  ABM39458  ABE47401  ADD91460  ZP_01786547  AAA79745  AAY78769  ABD28561  ABR74748  EAO61647  CAD59382  AAZ20279 |
| Pool 3 - hydrophilins | | | |
| Organism | | Accession number | |
| *Ashbya gossypii* | | NP_982805, NP_986053 | |
| *Caenorhabditis remanei* | | XP_003091135 | |
| *Candida albicans* | | XP_710966, XP_718825 | |
| *Candida dubliniensis* | | XP_002417291 | |
| *Candida glabrata* | | XP_445949, XP_447445, XP_448211 | |
| *Clavispora lusitaniae* | | XP_002619027 | |
| *Debaryomyces hansenii* | | XP_459742, XP_460524 | |
| *Kluyveromyces lactis* | | XP_452925 | |
| *Lachancea thermotolerans* | | XP_002551582 | |
| *Lodderomyces elongisporus* | | XP_001528079 | |
| *Meyerozyma guilliermondii* | | EDK40761, XP_001482904 | |
| *Saccharomyces cerevisiae* | | CAA92349, CAY77799, EDN60924, EDN62629, EGA84470, EGA56461, GRE1_YEAST, SIP18_YEAST, YBM6_YEAST, YJO4_YEAST, YNT0_YEAST | |
| *Scheffersomyces stipitis* | | XP_001382384 | |
| *Yarrowia lipolytica* | | XP_503076, XP_504184 | |
