# Supplementary material for: Comparison of Amino Acids Physico-Chemical Properties and Usage of Late Embryogenesis Abundant Proteins, Hydrophilins and WHy Domain
Source: PLoS One. 2014 Oct 8;9(10):e109570. doi: 10.1371/journal.pone.0109570 (PMC4190154; doi:10.1371/journal.pone.0109570)
Supplement: Table S2 — (DOC) [file pone.0109570.s006.doc]

**Table S2.** Three groups of parameters were used for the statistical analysis of the sequences: a group of 12 physico-chemical properties, a group of 20 relative counts of aminoacids and a group of 11 combination of plain percentages of aminoacids. This leads to a total of 43 properties.

| Physico-chemical properties | |
| --- | --- |
| MW/Length | Mean molecular weight |
| pI | Isoelectric point |
| Foldindex | Numerical prediction of intrinsic folding propensity [32] |
| Net charge | Mean net charge at pH 7 |
| Hydrophilicity | Mean hydrophylicity (scale: Hopp & Woods [33]) |
| GRAVY | Grand average of hydropathy (scale: Kyte & Doolittle [34]) |
| Hydrophobicity | Mean hydrophobicity (<H>) (scale: Eisenberg, Schwarz, Wall [35]) |
| Bulkiness | Mean bulkiness (scale: Zimmerman, Eliezer & Simha [36]) |
| Flexibility | Mean flexibility (scale: Bhaskaran & Ponnuswamy [37]) |
| Residues accessibility | Mean value of the molar fraction of 3220 accessible values per residue (scale: Janin [38]) |
| Buried residues | Mean value of the molar fraction of 2001 buried values per residue (scale: Janin [38]) |
| Transmembrane tendency | Mean transmembrane tendency value (scale: Zhao & London [39]) |
| Combinations of amino acids | |
| D+E | Fraction negative residues |
| K+R | Fraction positive residues |
| D+E+K+R | Fraction charged residues |
| D+E-K-R | Fractional net charge |
| A+I+L+V | Fraction hydrophobic residues |
| F+W+Y | Fraction aromatic residues |
| N+Q | Fraction amide residues |
| S+T | Fraction alcohol residues |
| C+W | Fraction rare / absent residues |
| R+E+S+P | Strong disorder promoting residues |
| C+F+Y+W | Strong order promoting residues |
